# Traumatic experiences and depression among Haitian caregivers: Evidence from the Grandi Byen study

**DOI:** 10.1017/gmh.2026.10257

**Published:** 2026-06-24

**Authors:** Michael Galvin, Rachel Beth Zimmerman, Gifty Aboagye-Mensah, Najjuwah Walden, Sherlie Jean Louis Dulience, Michelle Dorce, Patricia Kohl, Lora Iannotti

**Affiliations:** 1 Department of Behavioral Health Science and Practice, University of South Florida, USA; 2 E3 Nutrition Lab, School of Public Health, Washington University in St. Louis, USA; 3 School of Public Health, Washington University in St. Louis, USA; 4 Department of Social Work, Washington University in St. Louis School of Medicine, USA

**Keywords:** caregiver depression, traumatic exposure, humanitarian crisis, Haiti

## Abstract

This study investigates the relationship between trauma and caregiver depression in Haiti, a country burdened by ongoing political unrest, natural disasters, and economic hardship. A preponderance of evidence shows the substantial impact of caregiver mental health on child development and intergenerational vulnerability. This cross-sectional analysis examined data from the Grandi Byen randomized controlled trial, including 480 caregiver-infant dyads in Cap-Haitien. Depression risk was assessed using the Zanmi Lasante Depression Symptom Inventory (ZLDSI), and trauma exposure was measured with a survey adapted from the Life Events Checklist for DSM-5 (LEC-5). Negative binomial and ordinal logistic regression models assessed the relationship between caregiver trauma and depression, adjusting for demographic, socioeconomic, and environmental conditions. The analysis revealed that trauma exposure was significantly associated with higher odds of depression risk (OR = 1.09; 95% CI: 1.001, 1.193). Household composition was identified as a protective factor for depression (OR = 0.81; 95% CI: 0.664, 0.910). Trauma exposure was significantly associated with caregiver depression in Haiti, likely exacerbating the mental health challenges faced by caregivers in the context of political, economic and environmental stressors. Given the limited mental health data available in Haiti, this study provides essential insights into the trauma and challenges Haitians experience amidst ongoing crises.

## Impact statement

Caregiver mental health is a critical yet under-researched area in global public health, especially in fragile contexts like Haiti, where political instability, economic precarity, and environmental disasters intersect to create sustained trauma exposure. Within Haiti, there is a striking lack of recent mental health research, with Haitian studies currently contributing just 0.1% of regional mental health research output, leaving a significant gap in understanding the mental health challenges in this context. This study addresses that gap by investigating the relationship between trauma exposure and depression among Haitian caregivers, using data from a randomized controlled trial, Grandi Byen. Our findings reveal a significant link between trauma exposure and depression risk, while also highlighting household composition as a potential protective factor. These insights are especially critical in a setting where formal and informal mental health services are scarce, and caregivers often carry the compounded burdens of trauma, caregiving, and economic insecurity. By centering Haitian experiences within the global mental health landscape, this study provides an evidence base to support the development of culturally grounded, community-based interventions, as well as policy and resource allocation strategies that address the intergenerational impact of trauma and strengthen caregiver well-being in high-adversity environments.

## Introduction

Depression is a significant contributor to the global burden of disease and the primary cause of health-related disability (Liu et al., 2020). Approximately 4.7% of the global population experiences a depressive episode annually (Herrman et al., 2022; Lu et al., 2024). Despite its prevalence and impact, access to effective care remains alarmingly low, with over 80% of people in high-income countries and over 90% of people in low- and middle-income countries (LMICs) not receiving adequate care (Vigo et al., 2022). These disparities are particularly acute in Haiti, where recent crises have exacerbated mental health challenges faced by the population. While scientific data remains limited in this context, one study found mental, neurological, substance use disorders and suicide collectively account for 9% of all disability-adjusted life years in Haiti, highlighting their substantial health burden (Pan American Health Organization, 2020).

Trauma is a well-documented risk factor for depression. Its psychological consequences are often severe and enduring, with intergenerational impacts further compounding these effects (United States Center for Substance Abuse and Mental Health Services Administration, 2014). In Haiti, nuanced forms of trauma arise from complex and context-specific circumstances, including a history of political turmoil, natural disasters, limited resources, and persistent instability in the country. Recent overlapping crises in Haiti have placed caregivers, particularly mothers, under additional significant strain, exacerbating their already vulnerable position within Haitian society. Caregivers often bear a unique and compounded burden, shaped by persistent economic stress, entrenched gender inequalities and inadequate social support systems (Padgett and Warnecke, 2011; Cerdá et al., 2013; Pan American Health Organization, 2020; Singh et al., 2020; Usher et al., 2020; Lu et al., 2023; Rodrigues et al., 2023; El Khoury-Malhame et al., 2024). The intersection of these challenges not only heightens their susceptibility to depression but also has profound implications for their children’s well-being. Caregiver mental health is a critical determinant of child development, and the adverse effects of caregiver trauma and depression may ripple across generations, perpetuating cycles of vulnerability and hardship (Santos et al., 2024). These dynamics underscore the urgency of addressing the mental health needs of caregivers to safeguard both their well-being and that of their children.

Given the numerous humanitarian crises and environmental disasters in Haiti in recent years, research on mental health remains limited and insufficient, with Haitian studies contributing 0.1% of the regional mental health research output (Humanitarian Coalition, 2024). These persistent challenges – natural disasters, political instability, economic hardship, and other longstanding structural issues – have significantly worsened mental health issues in Haiti. Expanding research efforts is essential to comprehensively assess and respond to the mental health needs of the Haitian population. The current study addresses a critical gap by investigating the trauma-depression relationship in Haiti, providing much-needed evidence to inform interventions tailored to this context. This study aimed to (1) explore the risk of depression among caregivers in Haiti, (2) investigate the relationship between their traumatic experiences and risk of depression and (3) identify key factors influencing the relationship between trauma and depression within this context.

## Methods

This study is a cross-sectional, observational analysis of the baseline data from the *Grandi Byen* trial.

### Setting

The study activities were conducted in the Fort Saint Michel (FSM) Health Center in Cap-Haitien, Haiti. Cap-Haitien, the capital of the North Department (Nord), is the second largest city in Haiti after Port-au-Prince. FSM is located in Petite Anse, the city’s most impoverished commune, which is home to over 90,000 people. The area is densely populated and lies near a major road, a canal and an international airport. Due to its low-lying topography and inadequate water management infrastructure, the region is prone to flooding and mudslides during the rainy season. Healthcare access is limited, with one public hospital and two public clinics serving the population. The nearest mental health center, Sant Sante Mantal Mòn Pele, is ~7 km from FSM.

### Grandi Byen

*Grandi Byen* is a three-arm longitudinal randomized controlled trial evaluating the impact of an integrated nutrition, responsive parenting and WASH (water, sanitation and hygiene) intervention on holistic child growth and development. Although the primary focus of the trial was child growth and development, a mental health component assessing caregiver depression and trauma was incorporated into the approved study protocol at a later stage of implementation. Six hundred caregiver-infant dyads living in Cap-Haitien, Haiti, and in the surrounding communities of Petite Anse, FSM, and Madeleine were recruited and randomized into one of the following groups: (1) standard well-baby care; (2) nutritional intervention (one egg per day for 6 months); and (3) multicomponent *Grandi Byen* intervention (12-week responsive parenting, nutrition, WASH training and one egg per day for 6 months). More information regarding the protocol of this study can be found in the referenced paper (Kohl et al., 2022). Caregiver-infant dyads were recruited through referrals from community health agents, maternity and well-baby units at Fort Saint Michel clinic, and community rally posts. Caregivers were eligible to participate in the Grandi Byen Trial if they were at least 18 years of age and served as the primary caregiver of an infant between 6 and 8 months old. Dyads were excluded if infants exhibited congenital medical conditions, severe disabilities or severe acute malnutrition (defined as a weight-for-length *z*-score below −3). Additional exclusion criteria included known allergies to animal-source foods (including eggs, milk or fish) and being part of a multiple birth (*e.g.*, twin, triplet, *etc.*).

The mental health assessments were introduced beginning with the second cohort; the first cohort did not participate in depression or trauma screening. As a result, the present analyses include a subsample of 480 caregivers across four cohorts who completed the mental health measures. For clarity, these cohorts are referred to as Cohorts 1–4 in the present study, although they correspond to Cohorts 2–5 of the original Grandi Byen Trial. Eligible participants for the mental health portion of the study included adult caregivers responsible for the enrolled child’s daily care, including mothers, fathers, grandmothers and other relatives.

All participants provided written informed consent, which was amended before mental health data collection to include depression and trauma screening. Ethical approval for the Grandi Byen Trial, including the mental health component, was obtained from the Bioethics Committee of the Ministry of Public Health and Population (MSPP) in Haiti (#D) and the Institutional Review Board/Human Research Protection Office at Washington University in St. Louis (# 202101035; FWA00002284). This project is funded by the Eunice Kennedy Shriver National Institute of Child Health and Development (NICHD) (R01HD098255-02) and was registered on March 5, 2021, at https://clinicaltrials.gov/ (NCT04785352). Procedures were in place to ensure participant confidentiality, secure data storage and restricted access to data.

### Caregiver depression

The outcome for this analysis was caregiver depression. Depressive symptoms were measured by the Zanmi Lasante Depression Symptom Inventory (ZLDSI) (Rasmussen et al., 2015a; Legha et al., 2020). This 13-item screening tool identifies and assesses depressive symptoms using expressions of depression that are relevant to the context of Haiti (Supplementary Table S1). Each of the 13 items corresponds to a clinical depressive symptom such as “low energy,” “crying or feeling like crying” and “difficulty sleeping without waking early.” Items are scored on a 4-point scale (0–3), where 0 represents the absence of the symptom over the past 2 weeks, 1 indicates experiencing the symptom for 1–5 days, 2 for 6–9 days and 3 for 10–15 days. Total scores range from 0 to 39, with a score of 13 or higher indicating a threshold for potential depression, warranting further clinical evaluation or referral. Those participants who scored above the threshold were referred to the Mental Health Center at Morne Pelé. For ordinal regression models, depression scores were separated into four categories: 0–9 points, 10–19 points, 20–29 points and 30–39 points.

### Caregiver trauma

The primary predictor for this analysis was caregiver trauma. Caregiver trauma was measured through a survey adapted from existing trauma screeners, including the Life Events Checklist for DSM-5 (LEC-5) and informed by existing qualitative research of relevant traumatic events in Haiti (i.e., earthquakes and hurricanes) (Bolton and Gray, 2006; Grelotti et al., 2018). The 21-item survey includes 14 questions about specific traumatic experiences the caregiver may have encountered during their lifetime and 7 additional questions focused on traumatic experiences specifically related to or resulting from both the 2010 and 2021 earthquakes (Supplementary Table S2). Each item allowed the caregiver to respond whether they had experienced a particular event, and, if so, to rate how distressing it was. The response options were: “No,” “Yes, but no problem,” “Yes, a little problem,” “Yes, many problems” and “Yes, a big problem, and I couldn’t handle it.” For analysis, the total number of traumatic events experienced by each individual was summed. Small modifications were made based on the advice of local experts to be more relevant to northern Haiti, such as the removal of a question about combat or exposure to a war zone, as there is no war in this region. Other questions were added, such as being the victim of a mudslide – which is common in the hilly terrain of northern Haiti – or being the victim of a kidnapping, a common form of extortion by gangs in the region.

### Demographic, socioeconomic and environmental predictors

**Figure 1.**
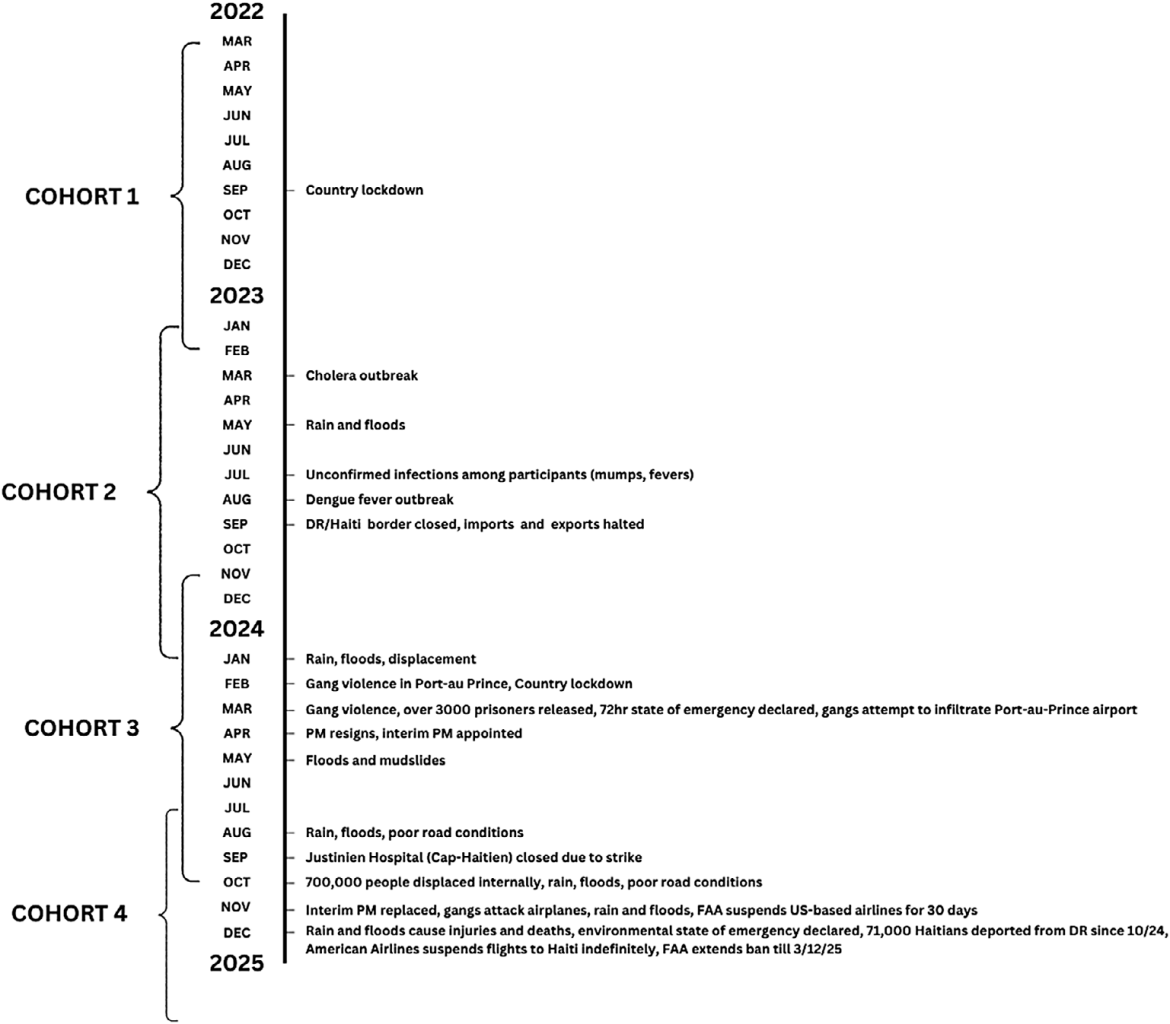
Timeline of mass traumatic events during the project period. Figure 1. long description.

Demographic, socioeconomic and environmental predictors regarded pertinent to control for after stepwise selection – a statistical method that iteratively adds or removes variables based on their contribution to the model’s explanatory power – include the caregiver’s age, caregiver’s education level, caregiver’s marital status, number of adults living in the caregiver’s household, caregiver’s home ownership status and study cohort. Participants of this study may have experienced traumatic events related to significant political or social upheaval, depending on the period they were involved in the study, as the participants’ period of involvement was defined by their cohort (Figure 1). Cohort 4 was specified as the reference group, as this group experienced the lowest average depression symptoms of the four cohorts.

### Statistical analyses

Caregiver characteristics were summarized and evaluated to provide context for the baseline population and identify variables relevant to the analyses. All statistical analyses were conducted in R 4.4.2. Bivariate linear models were employed to assess the association between caregiver depression and each individual predictor, while general linear regression models were used to examine the relationship between caregiver depression, caregiver trauma and the demographic, socioeconomic and environmental predictors mentioned above.

Analyses followed a two-stage approach. First, the number of traumatic events experienced by caregivers was modeled as an outcome to examine demographic, socioeconomic and cohort-related factors associated with trauma exposure. Due to overdispersion in Poisson regression models, negative binomial regression was used for this analysis (Cairney and Boyle, 2004).

Second, caregiver depression was modeled as an ordinal outcome, with traumatic events included as a key predictor alongside demographic, socioeconomic and environmental variables. Ordinal logistic regression was used to assess associations across increasing levels of depressive symptom severity. Stepwise selection was applied to identify these key variables for inclusion in all models. Diagnostics assessed collinearity and model fit using likelihood ratio tests and information criteria (Akaike and Bayesian Information Criteria [AIC and BIC, respectively]); overdispersion and residual diagnostics were evaluated for the negative binomial model, and the proportional odds assumption was assessed for the ordinal logistic model.

## Results

### Population characteristics

The sample includes 480 caregivers, with the majority being mothers (92.1%, 442 caregivers) and female (96.7%, 464 caregivers) (Supplementary Table S3). Two-thirds of caregivers were unmarried, with an average age of 29 years. Over three-quarters of caregivers completed secondary school. Caregivers averaged 2 days per week working outside the home. Households were typically composed of a few adults and children, and 64% of caregivers reported an income of 10,001 or more HTG, with a 62% receiving money transfers from abroad.

Homeownership was relatively uncommon; less than one-third of households owned their home, while the majority rented, shared or borrowed housing. Only 16% had access to electricity, and cooking practices primarily involved methods requiring more time or labor, such as cooking with charcoal or wood. The vast majority of homes had concrete floors at 83%, and a slight majority had aluminum or tin roofs. Water treatment was infrequent, and nearly three-quarters of households had outdoor toilets.

### Caregiver depression and trauma

The depression scores among the caregivers revealed that 14.8% of participants (71 caregivers) were identified as being at high risk for depression, scoring 13 or higher with ZLDSI (Table 1). The mean depression score (*x̄* = 5.87) across the population was below the threshold (13 or higher) in all cohorts (Cohort 1 *x̄* = 5.66; Cohort 2 *x̄* =6.40; Cohort 3 *x̄* = 6.10; Cohort 4 *x̄* = 5.33). The most common symptoms experienced among caregivers were “thinking too much” (49.8%, 239 caregivers), “feeling you have a constricted heart” (44.2%, 212 caregivers), “feeling tired or having little energy” (40.2%, 193 caregivers) and “low energy” (39.6%, 190 caregivers) (Supplementary Figure S1).

**Table 1.** Depression and trauma among the participant mothers Table 1. long description.

	(*n* = 480)
Depression score (SD)	5.87 (7.83)
Risk of depression (%)	
High risk of depression (depression score exceeding the cutoff)	14.8
Low risk of depression (depression score below the cutoff)	85.2
Number of traumatic events experienced (SD)	3.00 (2.50)
Type of event experienced (%)	
Motor or car accident	16.2
House fire	7.1
Hurricane or flood	35.6
Mudslide	3.54
Severe illness	11.5
Loss of livelihood	25.8
Robbed with a weapon	10.4
Rape	1.7
Witness a motor or car accident	24.4
Sudden death of a family member or close friend	38.5
Witness a person harmed	9.4
Witness a sudden death	14.8
Family member disappeared or was kidnapped	7.1
Earthquake	34
Neighborhood damaged	2.3
House destroyed	1
Serious injury	2.1
Death of a family member or close friend	14.2
Belongings lost	2.7
Forced to move	1.7
Housed displaced persons	1.5
Depression symptom experienced (%)	
Low energy	39.6
Feeling you have a constricted heart	44.2
Thinking too much	49.8
Crying or feeling like crying	33.5
Feeling you have lost the taste for doing anything	38.5
Feeling down, discouraged, or totally hopeless	37.5
Difficulty falling asleep	34.6
Feeling tired or having little energy	40.2
Having no appetite	30.4
Feeling you are a failure or feeling bad about yourself	31.7
Moving or speaking so slowly that people have noticed	24.6
Thoughts that you would be better off dead, or of hurting yourself in some way	18.3
Difficulty sleeping without waking early	25.6

A total of 395 caregivers reported experiencing at least one traumatic event, indicating that a significant proportion of the sample (~82%) had exposure to trauma. On average, participants reported approximately three traumatic events, reflecting a notable level of cumulative adversity within the group. The most common traumatic events experienced were the sudden death of a family member or close friend, hurricane or flood, earthquake and loss of livelihood (Table 1).

### Regression models: Caregiver trauma and depression

We used stepwise regression and the evidence base to test covariates with the potential for an association with trauma and depression. Those covariates showing significant associations and/or improving model fit were retained. The dyad characteristics assessed in these models, including the child-level characteristics, are reported as descriptives now in Supplementary Table S3.

The first model (Table 2) explored the relationship between traumatic events (dependent variable) and demographic and socioeconomic exposures (independent variables). Cohort 1 was the only statistically significant predictor of trauma among Grandi Byen mothers (OR = 0.888, 95% CI: [0.794, 0.992], *p* = .035), indicating that mothers in Cohort 1 had lower odds of experiencing traumatic events compared to the reference cohort. Caregiver characteristics, including age, education level, marital status and household factors such as home ownership and the number of adults in the home, were not significantly associated with trauma levels.

**Table 2.** Regression model for factors associated with trauma among Grandi Byen mothers Table 2. long description.

Coefficients	Estimate	Std. error	*p*-value	95% CI (Lower)	95% CI (Upper)
(Intercept)	2.88	0.257	0.0001	1.76	4.72
Cohort 1	0.888	0.0566	**0.0351**	0.794	0.992
Cohort 2	1.01	0.0326	0.657	0.952	1.08
Cohort 3	0.993	0.0231	0.768	0.949	1.04
Caregiver’s age	1.01	0.00610	0.0789	0.999	1.02
Caregiver’s education level	0.939	0.0412	0.129	0.868	1.02
Home ownership	0.947	0.0606	0.373	0.839	1.07
Number of adults in the home	1.01	0.0294	0.810	0.954	1.06
Caregiver’s marital status	0.997	0.0939	0.974	0.830	1.20

*Note:* Bold values are significant *p*-values (*p* > .05)

**Figure 2.**
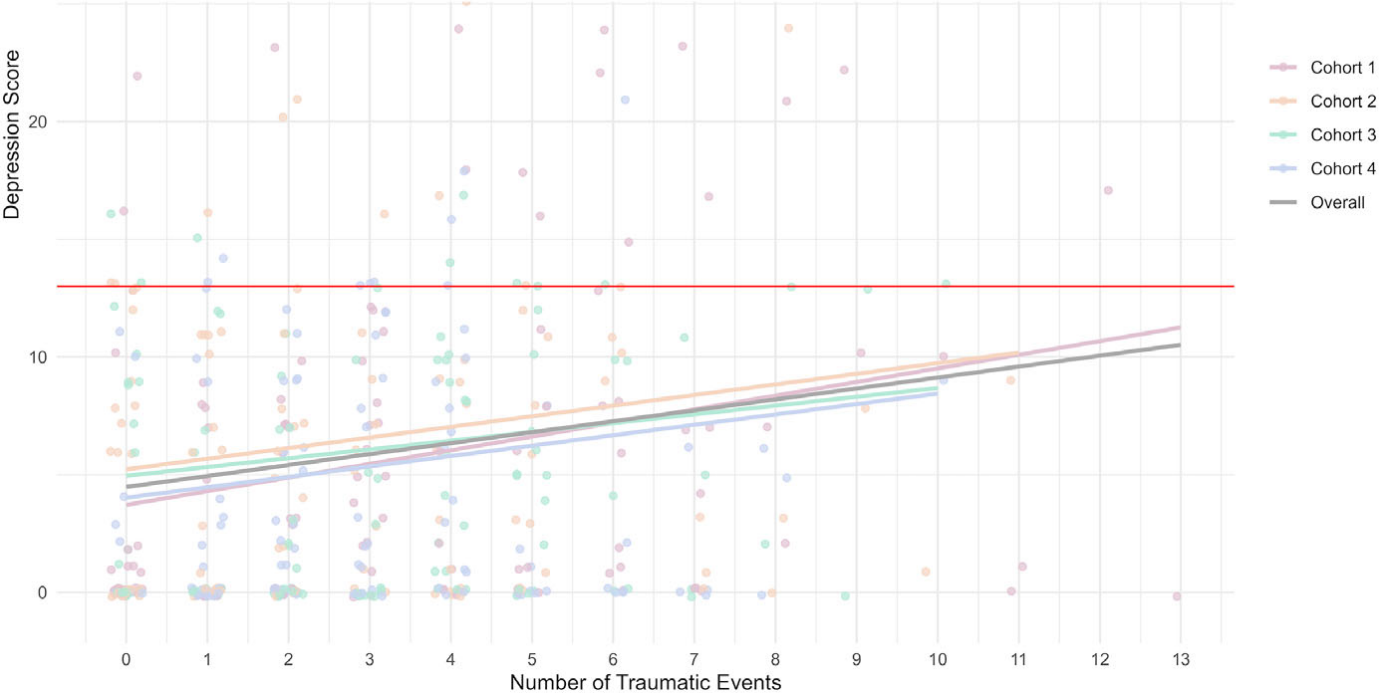
Relationship between depression risk and trauma across cohorts. Figure 2. long description.

The second model (Table 3) investigating the relationship between depression risk (outcome), trauma and demographic and socioeconomic predictors reveals that a higher number of traumatic events experienced is associated with an increased risk of depression. Cohort membership showed limited significant effects, with only one cohort exhibiting a marginally higher risk (Figure 2). Interactions between trauma and cohort membership were not associated with depression risk. A larger number of adults in the household was associated with a decreased depression risk. Caregiver characteristics, including age, education, marital status and home ownership, were not significantly associated with depression risk.

**Table 3.** Regression model for factors associated with depression risk among Grandi Byen mothers Table 3. long description.

Coefficients	Odds ratio	Std. error	*p*-value	95% CI (Lower)	95% CI (Upper)
Traumatic events	1.0932	0.0446	**0.0455**	1.0007	1.1925
Cohort 1	1.3765	0.2378	0.1791	0.8678	2.2160
Cohort 2	0.9980	0.1448	0.9889	0.7461	1.3194
Cohort 3	1.0282	0.1098	0.8002	0.8241	1.2700
Caregiver’s age	1.0026	0.0171	0.8777	0.9693	1.0365
Caregiver’s education level	0.9082	0.1136	0.3963	0.7241	1.1315
Home ownership	0.7662	0.1674	0.1116	0.5508	1.0629
Number of adults in the home	0.8135	0.0969	**0.0331**	0.6639	0.9695
Caregiver’s marital status	0.6557	0.2660	0.1126	0.3853	1.0961
Traumatic events*Cohort 1	0.9374	0.0543	0.2342	0.8412	1.0421
Traumatic events*Cohort 2	1.0169	0.0337	0.6188	0.9520	1.0869
Traumatic events*Cohort 3	0.9660	0.0303	0.2545	0.9081	1.0240

*Note:* Bold values are significant *p*-values (*p* > .05)

## Discussion

This study examined a large sample of primarily female Haitian caregivers living in extreme poverty, with little access to basic necessities such as electricity, gas for cooking, potable water, and indoor plumbing. Amidst a failing state and widespread insecurity, the vast majority of these caregivers had at least one traumatic event that significantly impacted their mental health, with an average of three per caregiver. Related to these traumatic events, this research also found relatively elevated levels of risk for depression among a sample of non-care-seeking caregivers, with many expressing both somatic and affective symptoms of depression. While some cohorts of this study exhibited higher rates of depression than others – potentially related to proximity to political violence in the country – overall models highlighted a strong relationship between traumatic experiences and depression, as well as poverty-related indicators – such as having a larger number of adults in the home – and depression.

Due to ongoing political and social crises in Haiti, few studies have been able to examine mental health among large population samples in recent years. Understanding the severity of the political and social upheaval that affected the lives of every Haitian is essential for the interpretation of this study’s findings. Recruitment for this study began 8 months after the assassination of Haitian President Jovenel Moïse on July 7, 2021, an event that set in motion the collapse of the state and the eventual takeover of large parts of the country by armed gangs. Moïse’s death was followed by the unelected appointment and resignation of Prime Minister Ariel Henry, and a 7.2 magnitude earthquake in the South Department (Diaz, 2021; Boulet-Groulx, 2022; United Nations Office for the Coordination of Humanitarian Affairs [OCHA], 2022). In addition to the political events, since March 2023, the country has also experienced torrential rainfall resulting in increased flooding, landslides, cholera, dengue fever and skin infections, in addition to being affected by hurricane Melissa last year (Crisis24, 2023; Chéry, 2024a, 2024b, 2024c, European Commission Humanitarian Aid and Civil Protection (ECHO), 2024; Pan American Health Organization (PAHO), 2023; Partners In Health (PIH), 2023).

Later in 2023, the Dominican Republic–Haiti border closed, and the Dominican Republic government suspended issuing visas to Haitians, preventing work and essential food and medicine supplies from crossing the border (Assessment Capacities Project (ACAPS), 2023; Famine Early Warning Systems Network (FEWS NET), 2023; Kanno-Youngs and Enecia Pérez, 2023). Since then, nearly 71,000 deportations from the Dominican Republic to Haiti have taken place, and nearly 1 in 2 Haitians reported experiencing hunger as of October 2024 (Enecia Pérez and Robles, 2024; World Food Programme (WFP), 2024). In response to political unrest, gang activity has also increased as a concerted effort to control Port-au-Prince, as well as other areas of the country, such as Artibonite (U.S. Committee for Refugees and Immigrants (USCRI), 2024). Gang attacks and barricades have caused many schools, hospitals and banks to close, and an estimated 700,000 people were internally displaced across the country (IOM, UN Migration, 2024; Sanon and Luxama, 2024). The consequences of these challenges have both directly and indirectly affected mental healthcare access, resulting in widespread suffering in the population (Cénat et al., 2025). As it has now been several years of unusually high levels of instability and lawlessness, impacts are compounded and affect the most vulnerable – notably women and children, as highlighted in this study – even more significantly.

This study’s assessment of mental health among 480 primarily female caregivers highlights a population that is largely very poor, with little formal education. Additionally, this is a sample of caregivers who were largely unmarried, therefore often struggling to provide for their children while living on a single income. Previous research in Haiti has found a strong link between poverty and mental health, underscoring the important role that poverty can play in factors that can lead to mental distress (Smith Fawzi et al., 2012; Wagenaar et al., 2012; Eugène, 2020; Cénat et al., 2022). In such conditions, social support can play a significant role in supporting single mothers, and having more adults in the household was found to be protective for depression in this study. This underlines the unique role that social support can play in bolstering mental health, particularly among unmarried female caregivers who often need help raising and supporting their young children. Particularly in the current context of Haiti, where the state has effectively ceased functioning, vulnerable individuals are most reliant on social support from friends and family members for survival. Additionally, several studies have stressed the importance of social support with regard to preventing depression among similar populations (Amédée et al., 2024; Hogan et al., 2024).

With regard to traumatic experiences, many of the caregivers reported events related to violence, displacement, and economic loss that influenced depression risk. Several studies have documented high levels of traumatic events among Haitians, and particularly among caregivers (Jaimes et al., 2008; Auguste and Rasmussen, 2019). A strong relationship between trauma and poor mental health has long been established in the scientific literature, further reinforced by the findings of this study (Martsolf, 2004; Belik et al., 2007; Byansi et al., 2023). In particular, a significant percentage of study participants reported being robbed with a weapon, having a family member kidnapped or disappeared or being the victim of rape, highlighting increases in crime and violence following the collapse of the state. In addition, over one-third said they had been the victims of a natural disaster such as a hurricane, flood, or earthquake. Lastly, over a quarter reported a loss of livelihood due to events outside their control. Previous mental health research in Haiti found similar rates of natural disaster and violence-related traumatic experiences (Bolton et al., 2012; Brewis et al., 2022). Overall, this study seeks to contribute to the limited mental health literature in Haiti that focuses on interventions for caregivers, with the goal of developing future longitudinal and mixed-methods research in this domain.

### Limitations and strengths

This study has several limitations. The data collection period overlapped with the onset of several significant periods of political and social upheaval, including a presidential assassination, environmental crises, and lockdowns. However, the current study does not examine how the timing of these events correlates with the onset of depressive symptoms. Instead, it focuses on the overall number of traumatic events and their contribution to differences in depression onset and severity. Given that data on depressive states among this population before these events is unavailable, this limitation highlights the need for future research that evaluates the impact of trauma on depression over time. Although we cannot assess changes before and after these events, understanding the duration of residual trauma is critical to understanding the progression of depression and the timeliness of depression treatment (Wang et al., 2023). As this study was quantitative in nature, it primarily focused on measurable outcomes, leaving a gap in understanding the unique emotional and physical experiences of each caregiver. A qualitative evaluation of psychological and psychosomatic symptoms could have provided deeper insights. Additionally, the study was limited in its ability to quantify distress or trauma severity, as no validated protocol exists for scoring the available response categories; consequently, we relied on event count alone, which may not capture differences in distress intensity.

In addition to limitations, this study also has several strengths. First, this research interviewed a relatively large sample of participants compared to previous studies in this region. Next, this study is important as it examined the mental health of primarily female caregivers in northern Haiti. Studies in Haiti and elsewhere around the world have shown significantly higher rates of depression among women compared to men – with mothers at even higher risk (Patel et al., 2002; Wagenaar et al., 2012; Rasmussen et al., 2015b). Therefore, this research focuses on an at-risk group with regard to mental health. Finally, while several studies have examined mental health in the south of Haiti (Hagaman et al., 2013; Blanc et al., 2016; Cadichon et al., 2017), only recent research by one study team has begun to examine it in the north of the country (Galvin et al., 2023). This research, therefore, fills an important gap in assessing mental health impacts in this region of Haiti.

## Conclusion

This study explored the impact of trauma on caregiver mental health in Haiti and revealed that trauma exposure is a major factor contributing to caregiver depression in Haiti, with significant implications for child development. Social support factors were identified as protective elements, suggesting that community and familial support can help mitigate some of the adverse effects of trauma. Given the limited mental health data available in Haiti, this study provides essential insights into the trauma and challenges that Haitians experience amidst ongoing crises. Additionally, this work has important clinical significance, as formal and informal mental health services are extremely limited. Ultimately, given the profound effects of caregiver depression on child development, interventions tailored to the socio-cultural context of Haiti are essential to addressing the long-term impact of trauma and improving outcomes for both caregivers and children.

## Supporting information

10.1017/gmh.2026.10257.sm001 Galvin et al. supplementary material Galvin et al. supplementary material

## Data Availability

Data collected for this project will be made available upon appropriate ethics/human subjects research approval and reasonable request from Grandi Byen’s principal investigators (LI and PLK), following the publication of the primary results from the trial. The authors anticipate that primary endpoint analyses will be completed within 24 months of endline data collection for the study’s final cohort.

## Supplementary material

The supplementary material for this article can be found at http://doi.org/10.1017/gmh.2026.10257.

## Long descriptions

### Figure 1. Long description

The timeline is structured along a central vertical line with years 2022, 2023, 2024, and 2025 marked.

On the left, four brackets define the study periods:

* COHORT 1: March 2022 to January 2023.

* COHORT 2: February 2023 to October 2023.

* COHORT 3: November 2023 to July 2024.

* COHORT 4: August 2024 to early 2025.

On the right, specific traumatic events are mapped to months:

* September 2022: Country lockdown.

* March 2023: Cholera outbreak.

* May 2023: Rain and floods.

* July 2023: Unconfirmed infections among participants (mumps, fevers).

* August 2023: Dengue fever outbreak.

* September 2023: D R forward slash Haiti border closed, imports and exports halted.

* January 2024: Rain, floods, displacement.

* February 2024: Gang violence in Port-au-Prince, Country lockdown.

* March 2024: Gang violence, over 3000 prisoners released, 72hr state of emergency declared, gangs attempt to infiltrate Port-au-Prince airport.

* April 2024: P M resigns, interim P M appointed.

* May 2024: Floods and mudslides.

* August 2024: Rain, floods, poor road conditions.

* September 2024: Justinien Hospital (Cap-Haitien) closed due to strike.

* October 2024: 700,000 people displaced internally, rain, floods, poor road conditions.

* November 2024: Interim P M replaced, gangs attack airplanes, rain and floods, F A A suspends U S-based airlines for 30 days.

* December 2024: Rain and floods cause injuries and deaths, environmental state of emergency declared, 71,000 Haitians deported from D R since 10 forward slash 24, American Airlines suspends flights to Haiti indefinitely, F A A extends ban till 3 forward slash 12 forward slash 25.

Navigate back to Figure 1..

### Table 1. Long description

The table presents data for n = 480 participants.

Section 1: Depression and Trauma Overview

- Depression score (S D): 5.87 (7.83).

- High risk of depression (above cutoff): 14.8%.

- Low risk of depression (below cutoff): 85.2%.

- Number of traumatic events experienced (S D): 3.00 (2.50).

Section 2: Type of Event Experienced (Percentage)

- Sudden death of a family member or close friend: 38.5%.

- Hurricane or flood: 35.6%.

- Earthquake: 34%.

- Loss of livelihood: 25.8%.

- Witness a motor or car accident: 24.4%.

- Motor or car accident: 16.2%.

- Witness a sudden death: 14.8%.

- Death of a family member or close friend: 14.2%.

- Severe illness: 11.5%.

- Robbed with a weapon: 10.4%.

- Witness a person harmed: 9.4%.

- House fire: 7.1%.

- Family member disappeared or was kidnapped: 7.1%.

- Mudslide: 3.54%.

- Belongings lost: 2.7%.

- Neighborhood damaged: 2.3%.

- Serious injury: 2.1%.

- Rape: 1.7%.

- Forced to move: 1.7%.

- Housed displaced persons: 1.5%.

- House destroyed: 1%.

Section 3: Depression Symptom Experienced (Percentage)

- Thinking too much: 49.8%.

- Feeling you have a constricted heart: 44.2%.

- Feeling tired or having little energy: 40.2%.

- Low energy: 39.6%.

- Feeling you have lost the taste for doing anything: 38.5%.

- Feeling down, discouraged, or totally hopeless: 37.5%.

- Difficulty falling asleep: 34.6%.

- Crying or feeling like crying: 33.5%.

- Feeling you are a failure or feeling bad about yourself: 31.7%.

- Having no appetite: 30.4%.

- Difficulty sleeping without waking early: 25.6%.

- Moving or speaking so slowly that people have noticed: 24.6%.

- Thoughts that you would be better off dead, or of hurting yourself in some way: 18.3%.

Navigate back to Table 1..

### Table 2. Long description

A statistical table with six columns: Coefficients, Estimate, Std. error, p-value, 95% C I (Lower), and 95% C I (Upper).

Data rows include:

* (Intercept): Estimate 2.88, Std. error 0.257, p-value 0.0001, C I 1.76 to 4.72.

* Cohort 1: Estimate 0.888, Std. error 0.0566, p-value 0.0351, C I 0.794 to 0.992.

* Cohort 2: Estimate 1.01, Std. error 0.0326, p-value 0.657, C I 0.952 to 1.08.

* Cohort 3: Estimate 0.993, Std. error 0.0231, p-value 0.768, C I 0.949 to 1.04.

* Caregiver’s age: Estimate 1.01, Std. error 0.00610, p-value 0.0789, C I 0.999 to 1.02.

* Caregiver’s education level: Estimate 0.939, Std. error 0.0412, p-value 0.129, C I 0.868 to 1.02.

* Home ownership: Estimate 0.947, Std. error 0.0606, p-value 0.373, C I 0.839 to 1.07.

* Number of adults in the home: Estimate 1.01, Std. error 0.0294, p-value 0.810, C I 0.954 to 1.06.

* Caregiver’s marital status: Estimate 0.997, Std. error 0.0939, p-value 0.974, C I 0.830 to 1.20.

Navigate back to Table 2..

### Table 3. Long description

The table contains six columns: Coefficients, Odds ratio, Std. error, p-value, 95% C I (Lower), and 95% C I (Upper).

* Traumatic events: Odds ratio 1.0932, Std. error 0.0446, p-value 0.0455, C I 1.0007 to 1.1925.

* Cohort 1: Odds ratio 1.3765, Std. error 0.2378, p-value 0.1791, C I 0.8678 to 2.2160.

* Cohort 2: Odds ratio 0.9980, Std. error 0.1448, p-value 0.9889, C I 0.7461 to 1.3194.

* Cohort 3: Odds ratio 1.0282, Std. error 0.1098, p-value 0.8002, C I 0.8241 to 1.2700.

* Caregiver’s age: Odds ratio 1.0026, Std. error 0.0171, p-value 0.8777, C I 0.9693 to 1.0365.

* Caregiver’s education level: Odds ratio 0.9082, Std. error 0.1136, p-value 0.3963, C I 0.7241 to 1.1315.

* Home ownership: Odds ratio 0.7662, Std. error 0.1674, p-value 0.1116, C I 0.5508 to 1.0629.

* Number of adults in the home: Odds ratio 0.8135, Std. error 0.0969, p-value 0.0331, C I 0.6639 to 0.9695.

* Caregiver’s marital status: Odds ratio 0.6557, Std. error 0.2660, p-value 0.1126, C I 0.3853 to 1.0961.

* Traumatic events times Cohort 1: Odds ratio 0.9374, Std. error 0.0543, p-value 0.2342, C I 0.8412 to 1.0421.

* Traumatic events times Cohort 2: Odds ratio 1.0169, Std. error 0.0337, p-value 0.6188, C I 0.9520 to 1.0869.

* Traumatic events times Cohort 3: Odds ratio 0.9660, Std. error 0.0303, p-value 0.2545, C I 0.9081 to 1.0240.

Navigate back to Table 3..

### Figure 2. Long description

The x axis is labeled Number of Traumatic Events, ranging from 0 to 13. The y axis is labeled Depression Score, ranging from 0 to over 20. A legend on the right identifies four groups: Cohort 1 in pink, Cohort 2 in orange, Cohort 3 in green, and Cohort 4 in blue, with a thick gray line representing the Overall trend.

* Data Distribution: Individual data points are plotted as semi-transparent dots colored by cohort. Most data points are clustered between 0 and 5 traumatic events and below a depression score of 10.

* Regression Lines: All four cohort lines and the overall gray line show a linear increase from left to right. Cohort 1 has the steepest positive slope, starting at a score of approximately 4 at 0 events and rising to 11 at 13 events. Cohort 4 has the lowest intercept and a shallower slope.

* Threshold: A solid red horizontal line is drawn across the entire graph at a Depression Score of approximately 13, serving as a clinical or risk cutoff. Only a small minority of individual data points across all cohorts fall above this red line.

Navigate back to Figure 2..
