# Supplementary material for: Traumatic experiences and depression among Haitian caregivers: Evidence from the Grandi Byen study
Source: Glob Ment Health (Camb). 2026 Jun 24;13:e139. doi: 10.1017/gmh.2026.10257 (PMC13373270; doi:10.1017/gmh.2026.10257)
Supplement: Galvin et al. supplementary material [file S205442512610257Xsup001.docx]

**Supplemental Table 1**: Zanmi Lasante Depression Symptom Inventory

|  | *Pandan 15 jou ki sòt pase la yo, konbyen fwa yon nan pwoblèm sa yo te fatige ou?*  (During the past 15 days, how many times did one of these problems bother you?) | Di tou | *Konbyen fwayon nan pwoblèm sa yo te fatige ou ?*  How many of these problems have tired you? | *Pandan kèk jou (1-5 jou)*  For a few days (1-5 days) | *Plis pase yon semèn (6--9 jou)*  More than a week (6-9 days) | *Preske chak jou (10-15 jou)*  Almost every day (10-15 days) |
| --- | --- | --- | --- | --- | --- | --- |
| 1 | *Santi ou de la la*  (Low energy) | 0 | --------- | 1 | 2 | 3 |
| 2 | *Santi kè sere*  (Feeling you have a constricted heart) | 0 | --------- | 1 | 2 | 3 |
| 3 | *Kalkile twòp*  (Thinking too much) | 0 | --------- | 1 | 2 | 3 |
| 4 | *Kriye oubyen anvi kriye*  (Crying or feeling like crying) | 0 | --------- | 1 | 2 | 3 |
| 5 | *Preske pa pran gou nan fè aktivite*  (Feeling you’ve lost the taste for doing anything) | 0 | --------- | 1 | 2 | 3 |
| 6 | *Santi ou kagou, dekouraje ak lavi, oubyen pèdi espwa nèt ale*  (Feeling down, discouraged or totally hopeless) | 0 | --------- | 1 | 2 | 3 |
| 7 | *Gen difikilte pou dòmi pran ou*  (Difficulty falling asleep) | 0 | --------- | 1 | 2 | 3 |
| 8 | *Santi ou fatige oubyen ou manke fòs*  (Feeling tired or having little energy) | 0 | --------- | 1 | 2 | 3 |
| 9 | *Ou pa gen apeti*  (Having no appetite) | 0 | --------- | 1 | 2 | 3 |
| 10 | *Ou santi lavi-w pase mal oubyen ou santi-w pa alèz ak tèt-w*  (Feeling you are a failure or feeling bad about yourself) | 0 | --------- | 1 | 2 | 3 |
| 11 | *Fè mouvman oubyen pale tèlman dousman, menm lòt moun wè sa*  (Moving or speaking so slowly that people have noticed) | 0 | --------- | 1 | 2 | 3 |
| 12 | *Ou di nan tèt ou: Pito-w te mouri, oubyen ou gen lide pou fè tèt-w mal*  (Thoughts that you would be better off dead, or of hurting yourself in some way) | 0 | --------- | 1 | 2 | 3 |
| 13 | *Gen difikilte pou dòmi san-w pa reveye bone*  (Difficulty sleeping without waking early) | 0 | --------- | 1 | 2 | 3 |

**Supplemental Table 2**: Trauma Survey

|  | *Non* (no) | *Wi men pat gen pwoblem* (yes but no problem) | *Wi, Yon ti pwoblem tou piti* (yes, a little problem) | *Wi, Pwoblem kelke pa* (yes, somewhat of a problem) | *Wi, Anpil pwoblem* (yes, many problems) | *Wi, Anpil pwoblem mwen pat ka sipote* (yes, big problem and I couldn’t handle it) |
| --- | --- | --- | --- | --- | --- | --- |
| *Eske ou pa janm fè pyès esperyans*___ (have you ever experienced ___): | | | | | | |
| 1. *Aksidan moto oubyen machin?* (motorcycle or car accident) |  |  |  |  |  |  |
| 2. *Kay ki pran dife?* (house fire) |  |  |  |  |  |  |
| 3. *Siklon ou inondasyon?* (hurricane or flood) |  |  |  |  |  |  |
| 4. *Glisman de teren?* (mudslide) |  |  |  |  |  |  |
| 5. *Ou te gen yon maladi grav?* (grave illness) |  |  |  |  |  |  |
| 6. *Pèdi tout ti aktivite ki te pèmèt ou viv?* (loss of livelihood) |  |  |  |  |  |  |
| 7. *Moun volè ou ak zam?* (robbed with a weapon) |  |  |  |  |  |  |
| 8. *Moun vyole ou?* (rape) |  |  |  |  |  |  |
| 9. *Temwen yon machin ou moto frape yon moun?* (witness a car or motocycle hit a person) |  |  |  |  |  |  |
| 10. *Mò sibit yon manm nan fanmi an ou zanmi pwòch?* (sudden death of a family member or close friend) |  |  |  |  |  |  |
| 11. *Temwen yon moun tire ou blese?* (witness a person shot or cut) |  |  |  |  |  |  |
| 12. *Temwen yon moun ki mouri sibit?* (witness a person die suddenly) |  |  |  |  |  |  |
| 13. *Manm fanmi ki disparet ou kinape?* (family member disappeared or kidnapped) |  |  |  |  |  |  |
| 14. *Tranblemandetè?* (earthquake) |  |  |  |  |  |  |
| *Eske ou pa janm fè esperyans pandan tranblemantè a* ________________________?  (During/due to the earthquake, did you experience ______?) | | | | | | |
| 1. *Viv nan yon zòn ki andomaje?* (lived in an area that was damaged?) |  |  |  |  |  |  |
| 2. *Kay ou te kraze?* (house was destroyed?) |  |  |  |  |  |  |
| 3. *Ou te blese trè grav?* (seriously injured?) |  |  |  |  |  |  |
| 4. *Manm fanmi ou oswa zanmi pwòch ou mouri?* (family member or close friend died?) |  |  |  |  |  |  |
| 5. *Pèdi preske tout byen ou?* (lost almost all your belongings?) |  |  |  |  |  |  |
| 6. *Ou te bezwen al abite nan yon lòt zòn?* (you had to move to another area?) |  |  |  |  |  |  |
| 7. *Ou te loje moun ki te gen pou deplase al viv nan lòt zòn?* (you had to house people who were displaced?) |  |  |  |  |  |  |

**Supplemental Table 3.** Characteristics of the sample (n=480)

|  |  |
| --- | --- |
| ***Caregiver Characteristics*** |  |
| Caregiver’s relation to the child (%) |  |
| Mother | 92.1 |
| Father | 3.3 |
| Grandmother | 1.5 |
| Other | 3.1 |
| Caregiver’s age, yrs (SD) | 29.2 (7.06) |
| Caregiver’s educational level (%) |  |
| Primary school | 24.0 |
| Secondary school | 54.2 |
| Vocational school | 4.0 |
| University | 13.8 |
| Graduate | 0.4 |
| None | 3.8 |
| Caregiver works outside of the home, days/wk (SD) | 2.09 (2.93) |
| Marital status (%) |  |
| Married | 32.5 |
| Unmarried | 67.5 |
| ***Household characteristics*** |  |
| Monthly household income (%) |  |
| 2500 – 5000 HTG (17.24 – 34.48 USD)^[[1]](#footnote-1)^ | 12.1 |
| 5001-7500 HTG (34.49 – 51.72 USD) | 9.0 |
| 7501-10,000 HTG (51.73 – 68.97 USD) | 15.4 |
| 10,001 HTG or more (68.98 USD or more) | 63.5 |
| Receives money transfer (%) | 61.9 |
| Adults living in household (SD) | 2.69 (1.35) |
| Children living in household (SD) | 2.90 (1.59) |
| Home ownership (%) | 26.5 |
| Land ownership (%) | 34.0 |
| Livestock production (%) | 29.8 |
| Electricity availability (%) | 16.4 |
| Cooking device (%) |  |
| Charcoal cooker | 80.4 |
| Gas stove (propane) | 12.3 |
| Gas stove (kerosene) | 6.7 |
| 3-stone (traditional) | 0.6 |
| Floor material (%) |  |
| Concrete | 83.1 |
| Ceramic | 8.1 |
| Earth/ground | 8.8 |
| House covering (%) |  |
| Concrete | 44.2 |
| Aluminum/tin | 55.8 |
| Water treatment (%) | 4.4 |
| Toilet location (%) |  |
| Outside of home | 72.5 |
| Inside of home | 27.5 |

**Supplemental Figure 1.** Depression severity of caregivers who experienced depression symptoms

**
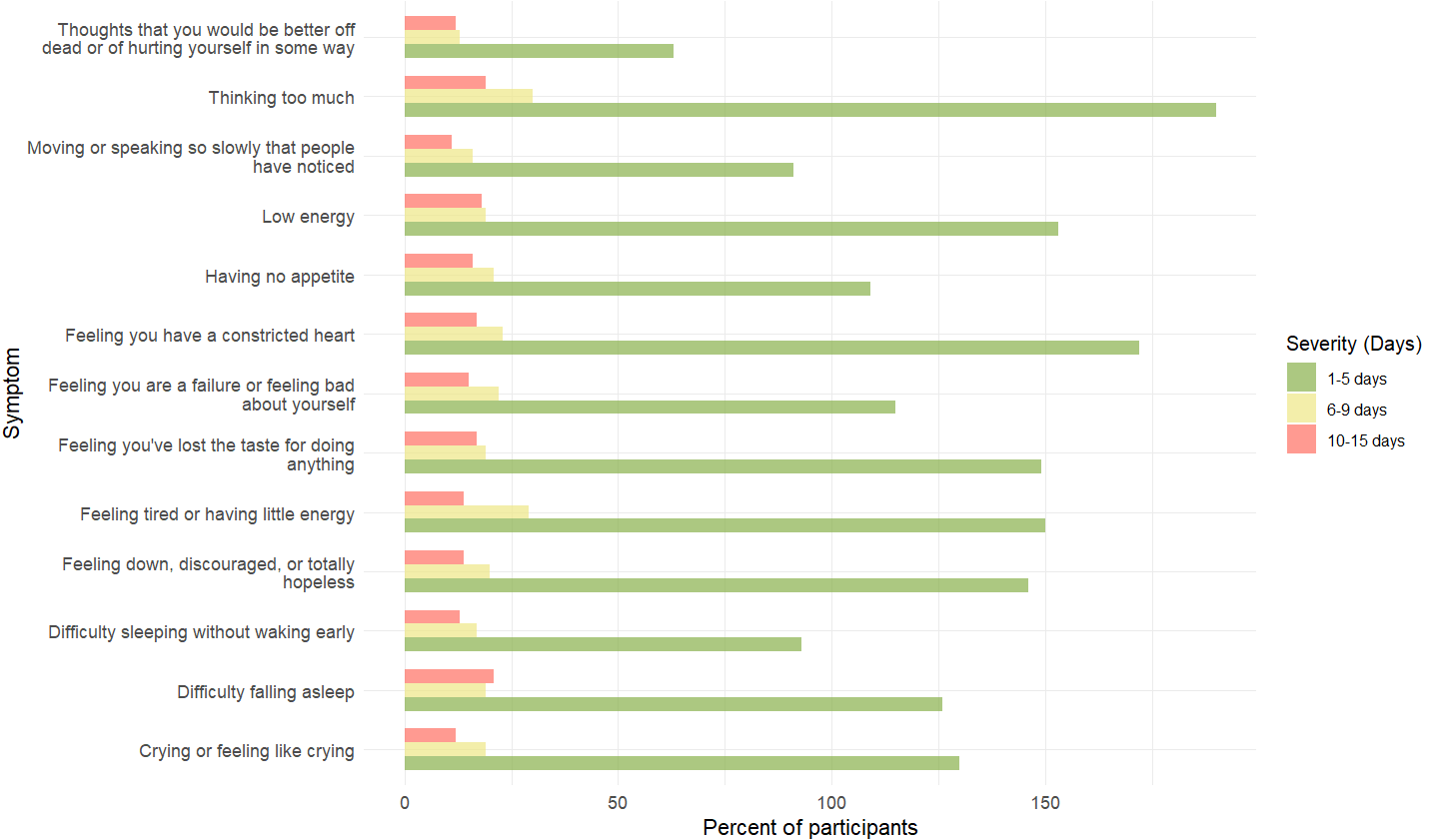
**

1. 145 HTG = 1 USD. Conversion based on 2022 receipt of in-field conversion, Cap-Haitien, Haiti. [↑](#footnote-ref-1)
