# Supplementary material for: Traumatic experiences and depression among Haitian caregivers: Evidence from the Grandi Byen study
Source: Glob Ment Health (Camb). 2026 Jun 24;13:e139. doi: 10.1017/gmh.2026.10257 (PMC13373270; doi:10.1017/gmh.2026.10257)
Supplement: Peer review history

# Reviewer report: Review: Traumatic experiences and depression among Haitian caregivers: Evidence from the Grandi Byen study — R0/PR2

**DOI:** 10.1017/gmh.2026.10257.pr2

Thank you for the opportunity to review this manuscript. It is timely, given the many structural challenges facing caregivers in Haiti. Some limitations need to be addressed to strengthen the manuscript.

1. Please include the consent procedures and ethical approval. Were any procedures in place for caregivers with clinical levels of depression? Did you provide any incentives for participation in the study? How were participants recruited in the main study?

2. For all the measures, it is essential to indicate their validity and reliability in this context. For the depression scale, what do the four categories correspond to or mean in terms of depression risk? 0-9 points, 10-19 points, and what is the clinical relevance of this categorization?

3. In the analytical approach, it is not clear why the negative binomial analysis was used on a predictor variable. Depression (ordinal variable) was the outcome, and traumatic events (count) were the predictor variable. The authors need to clarify why NBR was used in the analysis.

4. There is a typo on page 6, line 46. Additionally, please specify the currency for the income. In the population’s characteristics, the authors write “with a significant portion receiving money transfers.” This seems incomplete, from whom or where?

5. In the first model, page 7, line 18. “experiencing a greater number of traumatic events was significantly associated with higher trauma levels”. This sentence is confusing and not supported by Table 2. Please clarify?

6. In the discussion, the authors argue that they found a high risk for depression. Given the low mean (5), I would argue that this level is not high risk. The average in the sample is below the threshold of 13. I would suggest you tone down the language to reflect this overall statistic. It would be important to comment on the clinical significance of this work in the context of Haiti, where formal and informal mental health services are severely limited.

7. In the background, the authors document the impact of structural challenges on the mental health of caregivers. Although these are not measured, I would strongly suggest that the authors highlight how these impact overall health in Haiti specifically (poverty, natural disasters, political violence, etc.). The authors have primarily focused on political violence.

# Reviewer report: Review: Traumatic experiences and depression among Haitian caregivers: Evidence from the Grandi Byen study — R0/PR3

**DOI:** 10.1017/gmh.2026.10257.pr3

The strength of this paper is that it addresses a critical and underexplored public health issue by examining the relationship between trauma exposure and caregiver depression in Haiti, a setting marked by compounded political, environmental, and economic stressors. This research specifically contributes novel evidence to a severely limited body of mental health research in Haiti and offers valuable insights with implications for caregiver support, child development, and intergenerational health equity.

METHODS :

Although the authors make reference to the original protocol for the study, basic characteristics of the study sample should be presented in the methods section including participants Eligibility and inclusion criteria, age range, and caregiving role definitions.

Ethical Approval, local ethics approval in Haiti and Informed Consent procedures should be described in the methods section.

Lines 9- 19: The covariates / confounding variables section would benefit from having a description of the children’s characteristics in the dyad sample. Also, an explanation of Why specific covariates were selected could strengthen the paper.

Lines 32-56: Your measures are well chosen and contextually appropriate, but the section would benefit from greater methodological transparency. The authors alternate between “caregiver” and “maternal” depression/trauma. It is unclear whether all caregivers are mothers, or mothers are a subgroup of caregivers.

Specify the timeframe of measurement (Lifetime, adult-only, or since caregiving began) to establish causal interpretation in trauma–depression analyses.

Line 50: You state the trauma survey was “adapted” from the LEC-5 but do not describe how items were selected or modified; and whether local experts or pilot testing informed adaptation.

Scoring and Operationalization of Trauma, it is unclear why distress severity was collected but not used; and whether earthquake-related items were included in the total score or analyzed separately. A brief justification for using event count versus severity, and clarification of scoring decisions would help establish construction and analytic justification.

RESULTS: The Results section requires substantial revision to ensure that all analyses are clearly and consistently reported. Specifically, relevant statistical information should be explicitly incorporated for each analysis conducted. Strengthening the presentation of statistical results will improve clarity, transparency, and interpretability of the findings and ensure alignment with the journal reporting standards.

Line 46:

Check spelling for ”reporedt”.

STRENGTHS AND LIMITATIONS: The strengths of the study is described as part of the discussion section. However, the manuscript would benefit from the addition of clearly delineated Strengths and Limitations sections. Including these sections would strengthen the overall interpretation of the findings, align the manuscript with standard reporting practices, and enhance its credibility for both research and practice audiences.

DISCUSSION: The Discussion section provides a rich and well-documented contextual narrative that effectively situates the findings within Haiti’s complex political, environmental, and humanitarian landscape. A major strength of this section is its clear articulation of how cumulative trauma, poverty, and instability intersect to shape caregiver mental health, supported by extensive and relevant citations. That said, the Discussion would benefit from greater focus and synthesis to more clearly center the study’s primary findings. At times, the extensive background detail on political and environmental crises overshadows the interpretation of statistical results. Consider streamlining contextual descriptions and more explicitly linking them back to the key quantitative findings (e.g., trauma–depression associations, protective role of household composition). Additionally, the Discussion could be strengthened by more clearly distinguishing between results supported directly by the data and broader contextual interpretations drawn from the literature.

The section addressing traumatic experiences is informative but would benefit from greater analytic integration. Rather than listing frequencies of traumatic events, the authors could more explicitly discuss how specific categories of trauma (e.g., violence, displacement, economic loss) may differentially influence depression risk, even if explored descriptively. Similarly, while limitations related to timing, cross-sectional design, and lack of pre-crisis mental health data are acknowledged, these could be more succinctly framed and consolidated to avoid redundancy. Moreover, the Discussion would be strengthened by a clearer closing synthesis that emphasizes the study’s contribution to the limited mental health literature in Haiti, its implications for caregiver-focused interventions, and directions for future longitudinal and mixed-methods research.

Finally, some of the contextual information described in the Discussion would be better placed in a Background section, and a smaller portion could be streamlined or referenced in the Methods.

# Reviewer report: Review: Traumatic experiences and depression among Haitian caregivers: Evidence from the Grandi Byen study — R0/PR4

**DOI:** 10.1017/gmh.2026.10257.pr4

**Authors:** Youri Encelotti Louis

**Affiliations:** Social Medicine, EqualHealth, United States

Thank you for this valuable research. This study provides important data on the impact of traumatic events and associated depression risks in Global South countries facing complex situations. I particularly appreciate the use of culturally validated tools, such as the Zanmi Lasante Depression Symptom Inventory and the adapted Life Events Checklist for DSM-5.

After reviewing this first draft, I have the following questions and suggestions:

- Since the Grandi Byen trial focused on child growth and development rather than adult trauma, could you confirm that the approved protocol included a mental health component? I don’t seem to find it in the Grandi Byen Protocol. Please also confirm that participant consent was obtained for the collection and use of mental health data as part of the Grandi Byen Trial as well as how confidentiality and ethical access to data was part of the process.

- While the Grandi Byen Trial mentions 600 caregiver-infant dyads, this study includes 480 caregivers. Could you clarify how the inclusion criteria differed ?

- For ethical clarity, what interventions or follow-up resources were provided to the 14.8% of participants identified as having a high risk of depression?

- Given the focus on maternal mental health, the study would be more impactful if you highlighted data for female caregivers separately and provided the exact number of female participants.

- Finally, I believe a qualitative evaluation of symptoms would have further deepened the findings. But it’s already included as limitation.

Thank you again for your contribution to this field.

Best regards,

# Editor report: Recommendation: Traumatic experiences and depression among Haitian caregivers: Evidence from the Grandi Byen study — R0/PR5

**DOI:** 10.1017/gmh.2026.10257.pr5

**Affiliations:** Director, National Institute of Mental Health, Czech Republic

The reviewers have identified several shortcomings that need to be addressed. Please provide a response to each of the points raised.

# Editor report: Decision: Traumatic experiences and depression among Haitian caregivers: Evidence from the Grandi Byen study — R0/PR6

**DOI:** 10.1017/gmh.2026.10257.pr6

**Affiliations:** Johns Hopkins University Bloomberg School of Public Health, United States

No accompanying comment.

# Reviewer report: Review: Traumatic experiences and depression among Haitian caregivers: Evidence from the Grandi Byen study — R1/PR8

**DOI:** 10.1017/gmh.2026.10257.pr8

**Authors:** Youri Encelotti Louis

**Affiliations:** Social Medicine, EqualHealth, United States

The authors were very receptive to the feedback and suggestions provided to improve the article’s quality.

Great work detailing the research methodology. The models used and the statistical analysis appear impeccable. I am confident that this article provides essential mental health data to the Haitian scientific community that will effectively inform future strategies and programs.

# Reviewer report: Review: Traumatic experiences and depression among Haitian caregivers: Evidence from the Grandi Byen study — R1/PR9

**DOI:** 10.1017/gmh.2026.10257.pr9

The authors have addressed my previous comments. I have one comment on the discussion section (first paragraph, line 51). The authors indicate that poverty-related indicators were associated with depression. In tables 2 and 3, there are no findings to support this statement. Please clarify.

# Reviewer report: Review: Traumatic experiences and depression among Haitian caregivers: Evidence from the Grandi Byen study — R1/PR10

**DOI:** 10.1017/gmh.2026.10257.pr10

Thank you for your attention to the previous reviewer comments and for addressing several of the suggested revisions. This manuscript addresses a critical and underexplored public health issue by examining the relationship between trauma exposure and caregiver depression in Haiti, contributing valuable evidence to a limited body of mental health research in this context. The following comments are intended to further strengthen the methodological transparency, reporting of results, and overall organization of the manuscript.

Methods

Ethical Approval:

Please include the Federal Wide Assurance (FWA) number associated with the Institutional Review Board (IRB) that approved this study. Providing this information is necessary to ensure compliance with ethical reporting standards.

Caregiver Trauma Section:

While a prior comment was partially addressed by stating that “small modifications were made based on the advice of local experts to be more relevant to northern Haiti,” the manuscript still does not sufficiently describe what those modifications entailed. For example, the trauma survey is described as being adapted from the LEC-5; however, the process by which items were selected, modified, or omitted is not explained. Please provide additional detail regarding how the instrument was adapted for this population and specify what “small modifications” were made to improve cultural relevance.

Results: The Results section would benefit from substantial revision to ensure that analyses are clearly and consistently reported. Relevant statistical information should be explicitly incorporated for each analysis conducted. Strengthening the presentation of statistical results will improve clarity, transparency, and interpretability of the findings, as well as ensure alignment with journal reporting standards.

For example, all reported percentages should be accompanied by the corresponding sample sizes. Additionally, the statement that “the mean depression score across the population was below the threshold in all cohorts” is presented without supporting statistical values. Please provide the relevant descriptive statistics (e.g., means and standard deviations) to support this assertion.

Similarly, the statement that “the most common symptoms experienced among caregivers were ‘thinking too much,’ ‘feeling you have a constricted heart,’ ‘feeling tired or having little energy,’ and ‘low energy’” is not accompanied by any statistical data. Please report the frequency or proportion of participants endorsing these symptoms.

The statement that “Cohort 1 showed experiencing a notably significantly lower estimated amount of traumatic events compared to other cohorts” also requires supporting statistical information. Please provide the appropriate comparative statistics to demonstrate differences between Cohort 1 and the other cohorts.

Discussion: The primary focus of the Discussion section should be on interpretation of the study findings. Currently, this section includes six paragraphs, but only one directly addresses the results of the study. It would be beneficial to strengthen the Discussion by providing a more balanced interpretation of the findings in relation to the existing literature, rather than focusing predominantly on the broader context of sociopolitical unrest in Haiti. Additionally, some of the contextual information included in the Discussion may be more appropriately placed in the Background section. A portion of this material could also be streamlined or referenced in the Methods section where relevant.

# Editor report: Recommendation: Traumatic experiences and depression among Haitian caregivers: Evidence from the Grandi Byen study — R1/PR11

**DOI:** 10.1017/gmh.2026.10257.pr11

**Affiliations:** Director, National Institute of Mental Health, Czech Republic

Please respond and address all of the reviewers' comments.

# Editor report: Decision: Traumatic experiences and depression among Haitian caregivers: Evidence from the Grandi Byen study — R1/PR12

**DOI:** 10.1017/gmh.2026.10257.pr12

**Affiliations:** Johns Hopkins University Bloomberg School of Public Health, United States

No accompanying comment.

# Reviewer report: Review: Traumatic experiences and depression among Haitian caregivers: Evidence from the Grandi Byen study — R2/PR14

**DOI:** 10.1017/gmh.2026.10257.pr14

Thank you for your attention to the previous comments and for addressing many of the comments. This manuscript addresses a critical and underexplored public health issue by examining the relationship between trauma exposure and caregiver depression in Haiti, contributing valuable evidence to a limited body of mental health research in this context. The following comments are intended to further strengthen the methodological transparency, and reporting of results of the manuscript.

Methods:

Caregiver Trauma Section: The following statement is not necessary and can be removed; this information is already well articulated in the methods section “The most significant changes were the 7 additional questions specific to the 2010 and 2021 earthquakes which caused significant disruption and hardship across the country”

While a prior comment was partially addressed by stating that “small modifications were made based on the advice of local experts to be more relevant to northern Haiti,” the manuscript still does not sufficiently describe what those modifications entailed?

Results:

The Results section would benefit from minor revisions to ensure that analyses are clearly and consistently reported. It is not clear what is considered “Most” or “Many” – The following statements should be supported by the related statistics.

Line 34 -Most caregivers were unmarried………….

Line 37 - Many caregivers worked outside the home, but most worked only a few days per week….

Line 38 - Most caregivers reported an income….

Line 44- Most do not have access to electricity…...

Line 46 - Most homes had concrete floors……

Lines 35-43- There are no statistics reported to support the claims.

# Editor report: Recommendation: Traumatic experiences and depression among Haitian caregivers: Evidence from the Grandi Byen study — R2/PR15

**DOI:** 10.1017/gmh.2026.10257.pr15

**Affiliations:** Director, National Institute of Mental Health, Czech Republic

Please address all points raised by the reviewer.

# Editor report: Decision: Traumatic experiences and depression among Haitian caregivers: Evidence from the Grandi Byen study — R2/PR16

**DOI:** 10.1017/gmh.2026.10257.pr16

**Affiliations:** Johns Hopkins University Bloomberg School of Public Health, United States

No accompanying comment.

# Editor report: Recommendation: Traumatic experiences and depression among Haitian caregivers: Evidence from the Grandi Byen study — R3/PR18

**DOI:** 10.1017/gmh.2026.10257.pr18

**Affiliations:** Director, National Institute of Mental Health, Czech Republic

Thank you for this valuable contribution to the field of global mental health.

# Editor report: Decision: Traumatic experiences and depression among Haitian caregivers: Evidence from the Grandi Byen study — R3/PR19

**DOI:** 10.1017/gmh.2026.10257.pr19

**Affiliations:** Johns Hopkins University Bloomberg School of Public Health, United States

No accompanying comment.
